# Supplementary material for: Bacterial evolution of antibiotic hypersensitivity
Source: Mol Syst Biol. 2013 Oct 29;9:700. doi: 10.1038/msb.2013.57 (PMC3817406; doi:10.1038/msb.2013.57)
Supplement: Supplementary Information [file msb201357-s1.doc]

Supplementary Information

Bacterial evolution of antibiotic hypersensitivity

Viktória Lázár, Gajinder Pal Singh, Réka Spohn, István Nagy, Balázs Horváth, Mónika Hrtyan, Róbert Busa-Fekete, Balázs Bogos, Orsolya Méhi, Bálint Csörgő, György Pósfai, Gergely Fekete, Balázs Szappanos, Balázs Kégl, Balázs Papp & Csaba Pál

Correspondence should be sent to either C.P. ([cpal@brc.hu](mailto:cpal@brc.hu)) or B.P. ([pappb@brc.hu](mailto:cpal@ramet.elte.hu?subject=email subject)).

Table of contents

[Supplementary Figures 3](#__RefHeading___Toc367723123)

[Supplementary Figure S1 Confirming fitness measurements 3](#__RefHeading___Toc367723125)

[Supplementary Figure S2 Parallel evolving populations exposed to the same antibiotic show very similar antibiotic susceptibility patterns. 5](#__RefHeading___Toc367723126)

[Supplementary Figure S3 Collateral sensitivity is not due to cost of resistance under antibiotic-free medium. 6](#__RefHeading___Toc367723128)

[Supplementary Figure S4 Membrane potential changes in populations adapted towards gradually increasing antibiotic concentrations. 7](#__RefHeading___Toc367723129)

[Supplementary Figure S5 Membrane permeability (Hoechst dye) changes in populations adapted towards gradually increasing antibiotic concentrations. 8](#__RefHeading___Toc367723130)

[Supplementary Figure S6 Optimization of the high-throughput fitness measurement assay. 9](#__RefHeading___Toc367723131)

[Supplementary Tables 11](#__RefHeading___Toc367723132)

[Supplementary Table S1 Estimated MIC changes following the laboratory evolution towards gradually increased antibiotic dosage 11](#__RefHeading___Toc367723133)

[Supplementary Table S2 Raw dataset of collateral sensitivity interactions identified at the level of antibiotic pairs. 12](#__RefHeading___Toc367723134)

[Supplementary Table S3 Decrease in minimum inhibitory concentrations of aminoglycoside-adapted lines. 12](#__RefHeading___Toc367723135)

[Supplementary Table S4 SNPs identified in aminoglycoside-adapted populations. 15](#__RefHeading___Toc367723136)

[Supplementary Table S5 Genes/protein complexes mutated multiple times in aminoglycoside adapted lines. 16](#__RefHeading___Toc367723137)

[Supplementary Table S6 Functional enrichment analysis of SNPs. 19](#__RefHeading___Toc367723138)

[Supplementary Table S7 Pleiotropic effects of a single mutation in trkH 20](#__RefHeading___Toc367723139)

[Supplementary Table S8 Collateral sensitivity data on populations adapted to fixed, sublethal antibiotic concentrations 21](#__RefHeading___Toc367723140)

[Supplementary Table S9 Collateral sensitivity data on populations adapted to gradually increased antibiotic concentrations 21](#__RefHeading___Toc367723141)

[Supplementary Texts 22](#__RefHeading___Toc367723142)

[Supplementary Text S1 Processing of high-throughput bacterial growth data 22](#__RefHeading___Toc367723143)

[Supplementary Figure S7 Regression modelling of spatial effects in high-throughput growth assays. 23](#__RefHeading___Toc367723144)

[Supplementary Figure S8 Normalization procedures reduce measurement bias and variation of control plate experiments. 25](#__RefHeading___Toc367723145)

[Supplementary Text S2 Accuracy of high-throughput interaction measurements and control for potential confounding factors 26](#__RefHeading___Toc367723146)

[References 28](#__RefHeading___Toc367723147)

# Supplementary Figures


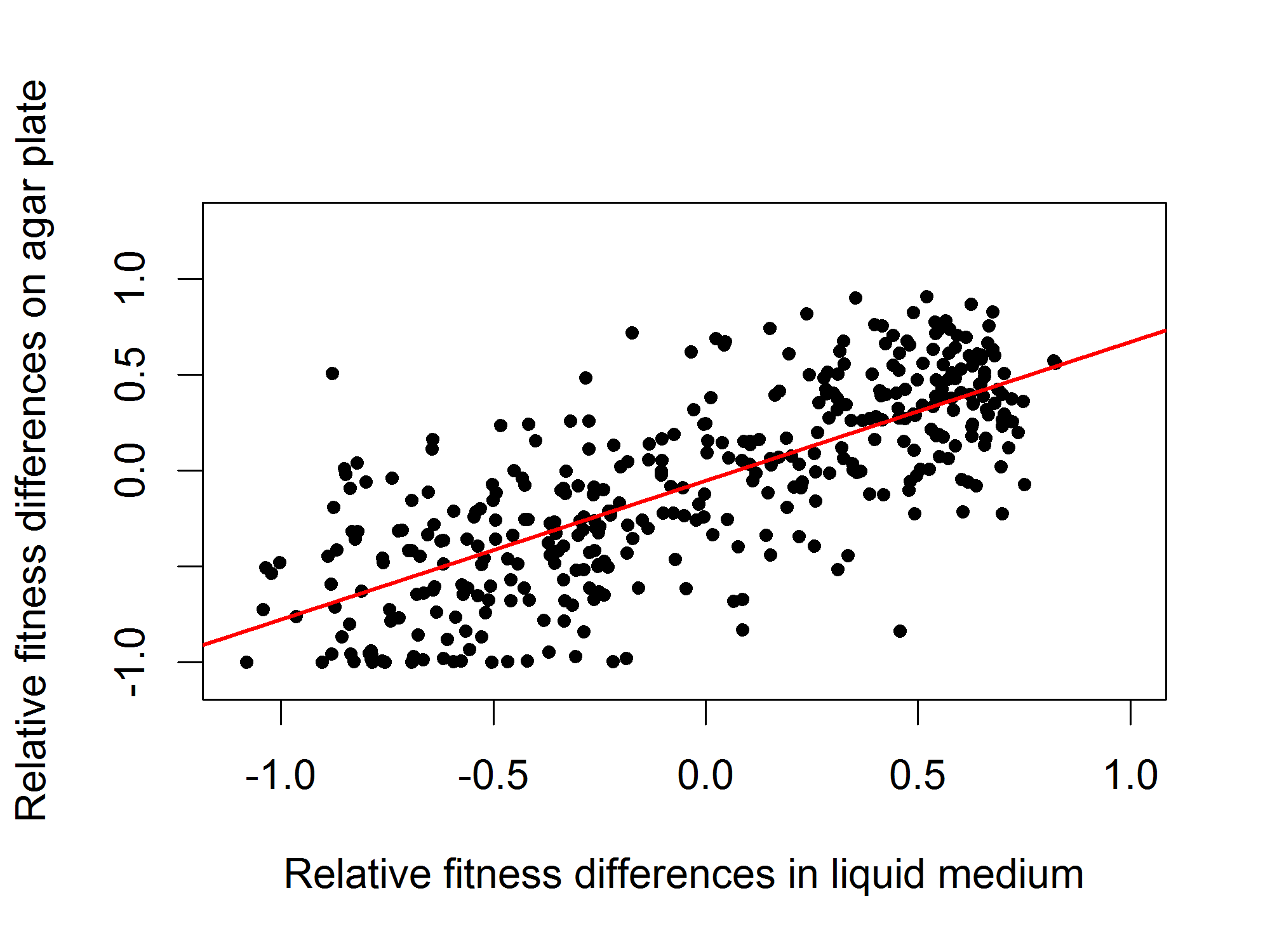


## Supplementary Figure S1 Confirming fitness measurements

Relative fitness in liquid medium and on agar plates show strong correlation (Spearman’s rho = 0.75, P= 10^-16^, N=392). To validate the reliability of our high-throughput protocol, fitness was also estimated by colony size on agar plates supplemented with 14 different antibiotics for a set of representative laboratory-evolved strains (28 in three replicates). A custom-built pipeline was used to quantify colony size, using CellProfiler (7). Relative fitness differences indicate comparisons of evolved and wild-type populations.

a)


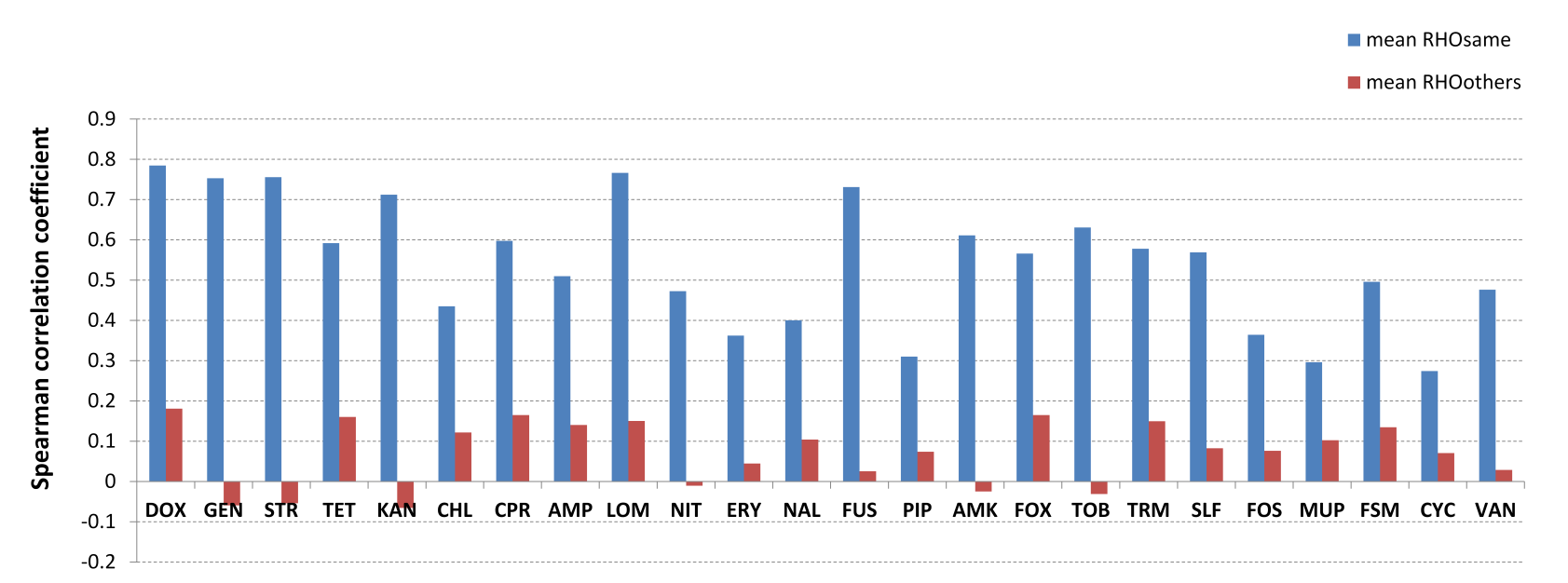


b)


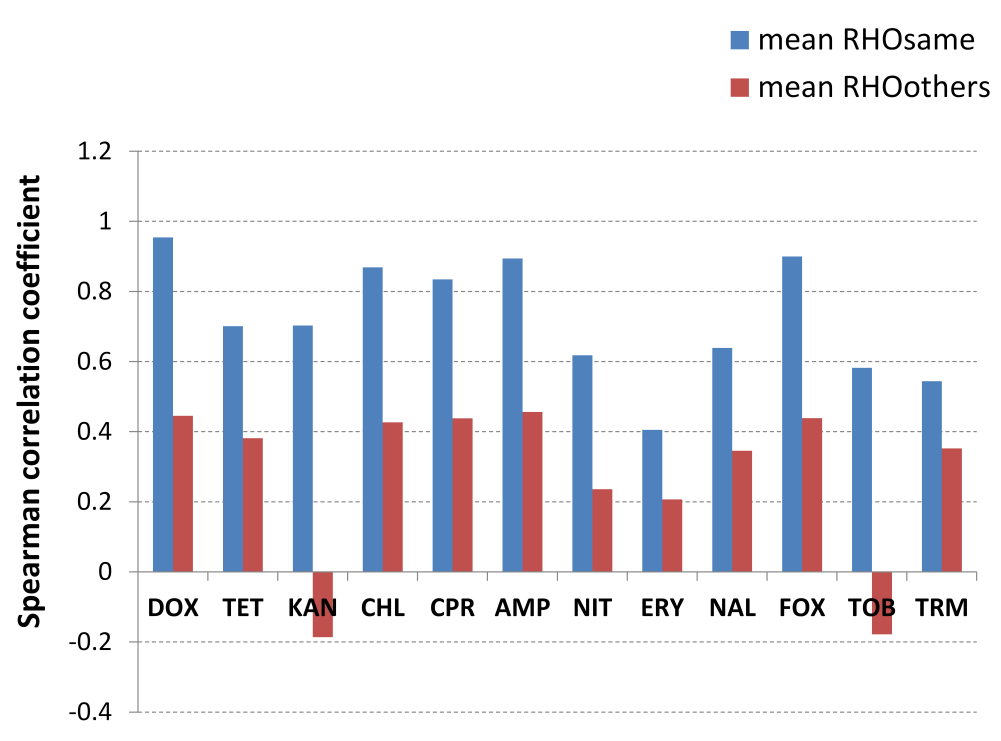


## Supplementary Figure S2 Parallel evolving populations exposed to the same antibiotic show very similar antibiotic susceptibility patterns.

Results from populations adapted to a) fixed, sublethal antibiotic concentrations, and b) gradually increased antibiotic dosages.

The mean relative fitness differences (RF) were computed between the adapted control and the antibiotic evolved line in the presence of each antibiotic (Text S2). This dataset allows comparisons of RF profiles for all possible pairwise combinations of laboratory evolved populations. The calculated Spearman correlation coefficients were significantly higher between pairs of populations exposed to the same antibiotic (RHOsame) than between pairs of populations exposed to different antibiotics (RHOothers). Wilcoxon signed rank test, FDR adjusted P values were <0.0005 in all cases. For antibiotic abbreviations, see Table 1, main text.


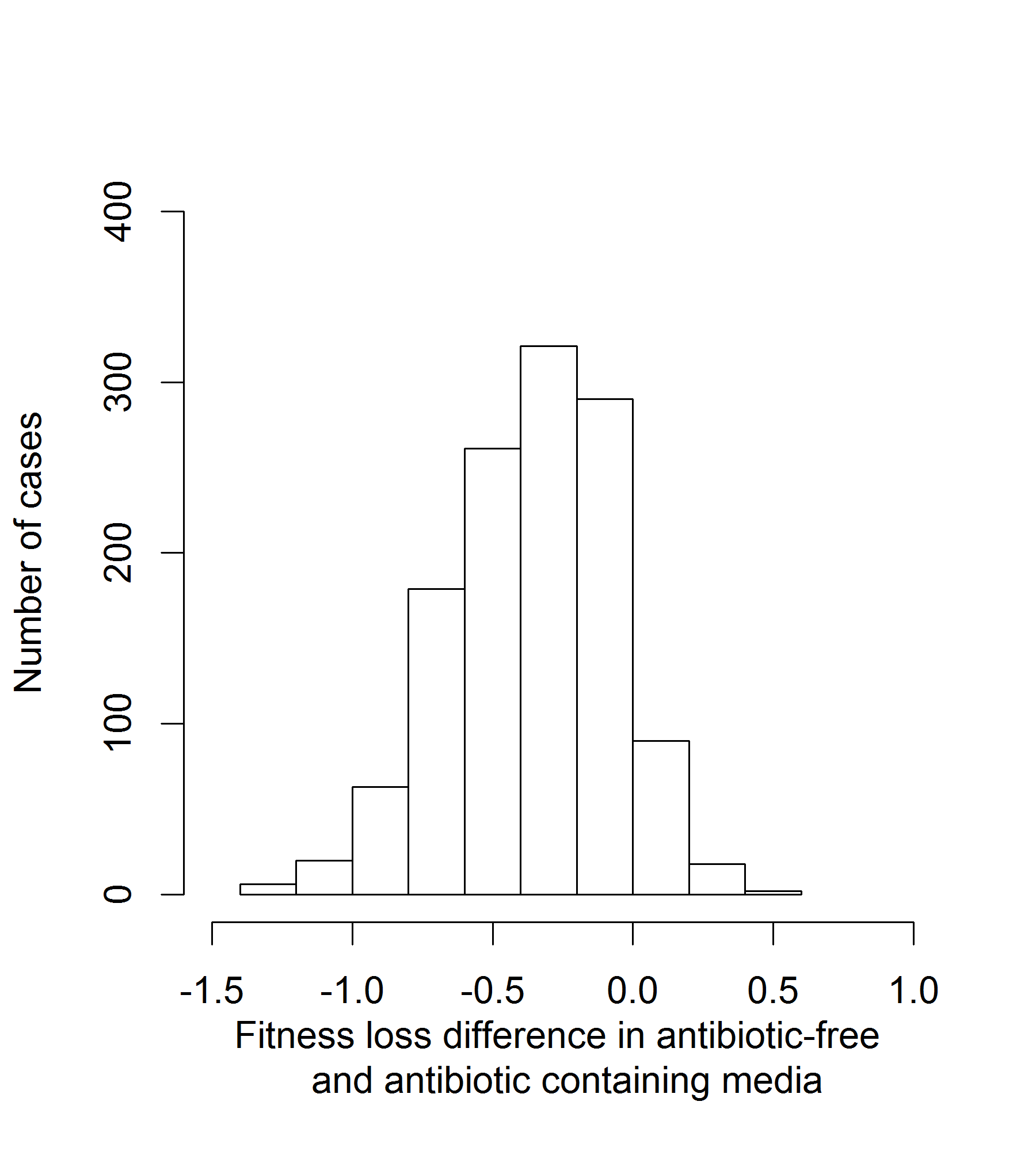

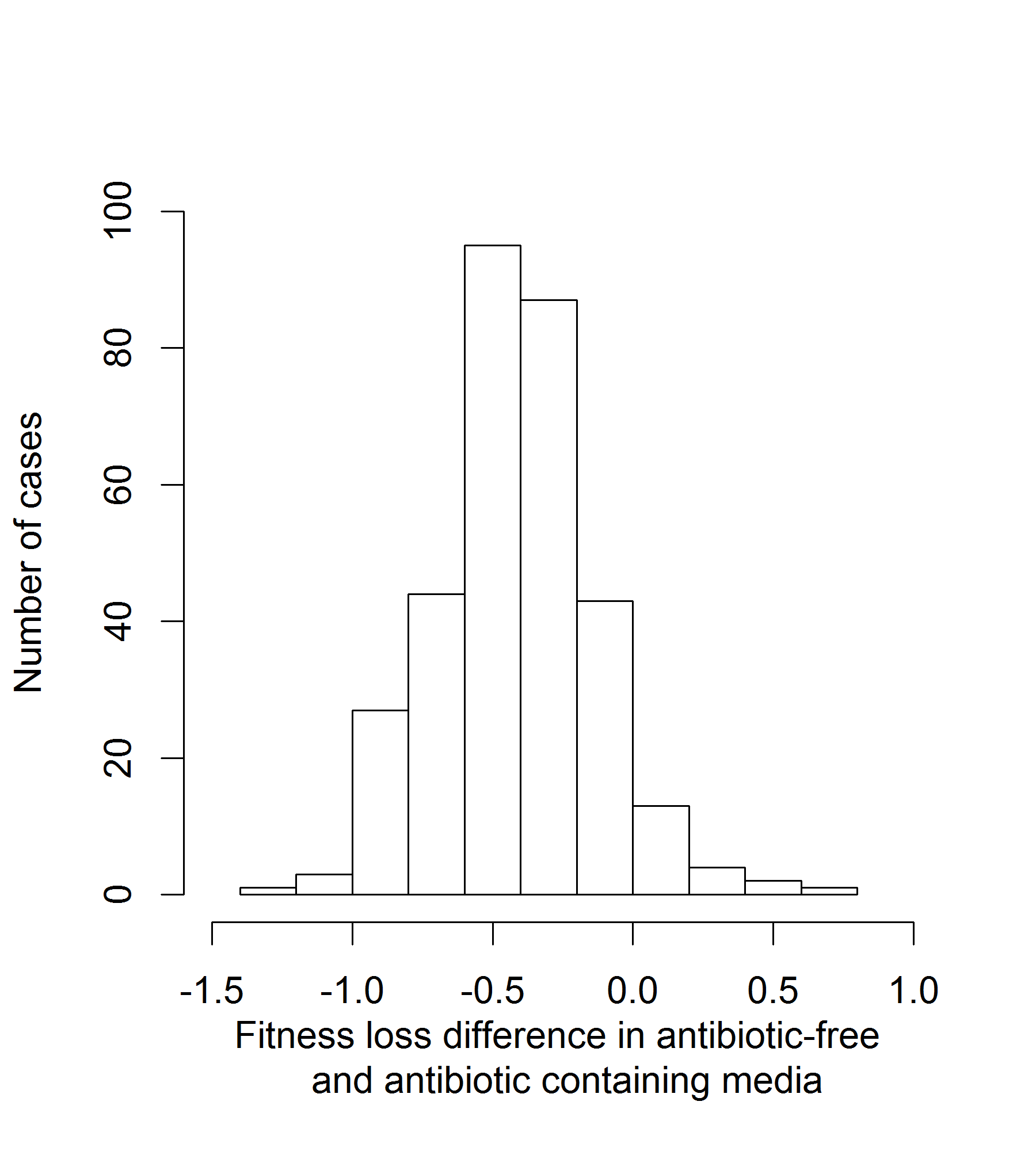


## Supplementary Figure S3 Collateral sensitivity is not due to cost of resistance under antibiotic-free medium.

Distribution of the differences between fitness loss in antibiotic-containing (collateral sensitivity) and antibiotic-free media (general cost of resistance). Panel a) and b) show results with strains adapted to constant and gradually increasing dosages, respectively. By comparing fitness of antibiotic-adapted and adapted control populations on both antibiotic-free and antibiotic-containing media, we could discriminate hypersensitivity to an antibiotic from general cost of resistance (see Supplementary Text S2 and data available in Supplementary Tables S8-S9). The figures demonstrate that the growth defect associated with collateral sensitivity to specific antibiotic-containing medium was generally much higher than the observed cost of resistance of particular antibiotic-adapted populations in antibiotic-free medium.

a)

b)


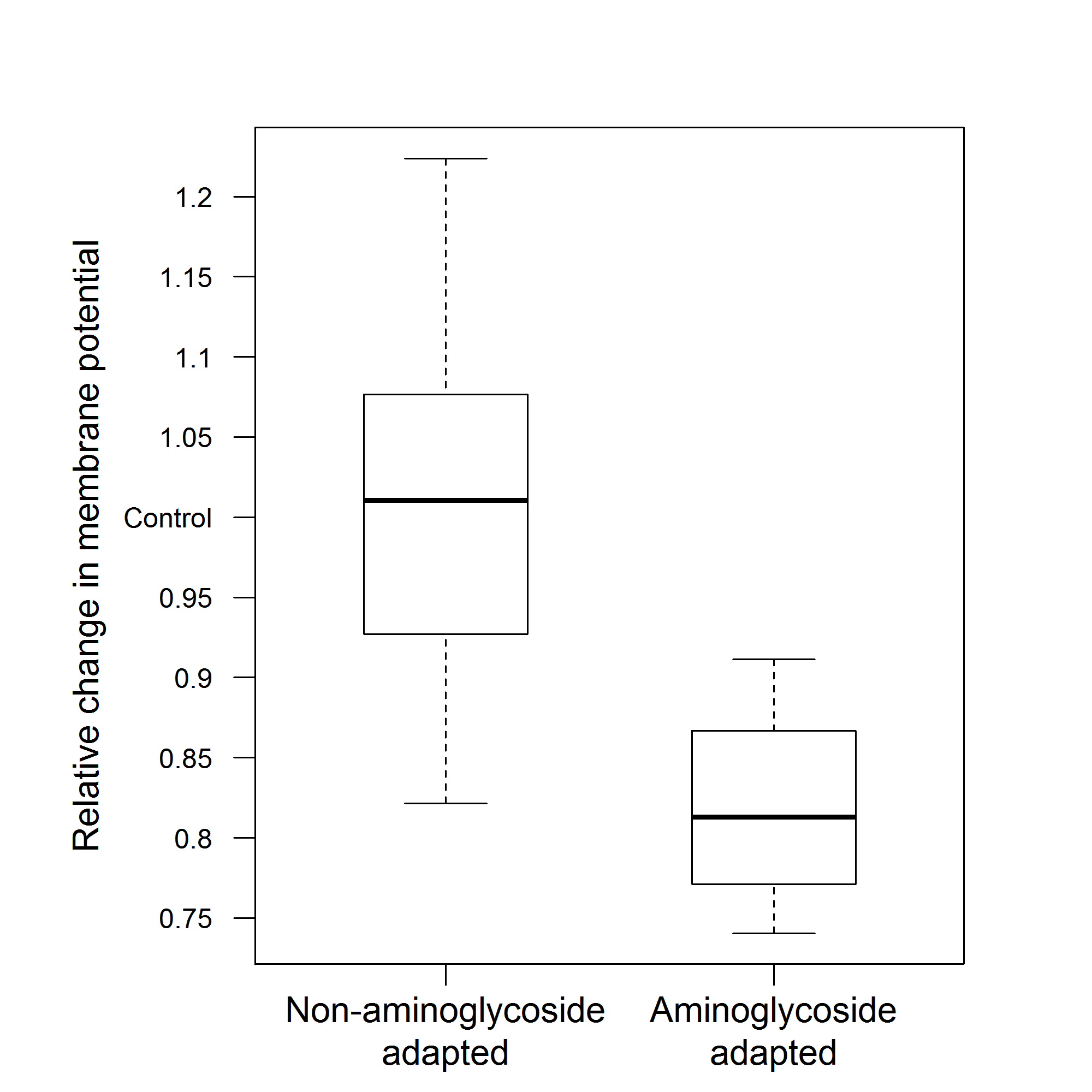


## Supplementary Figure S4 Membrane potential changes in populations adapted towards gradually increasing antibiotic concentrations.

For details, see Materials and Methods and Figure 4A, main text.


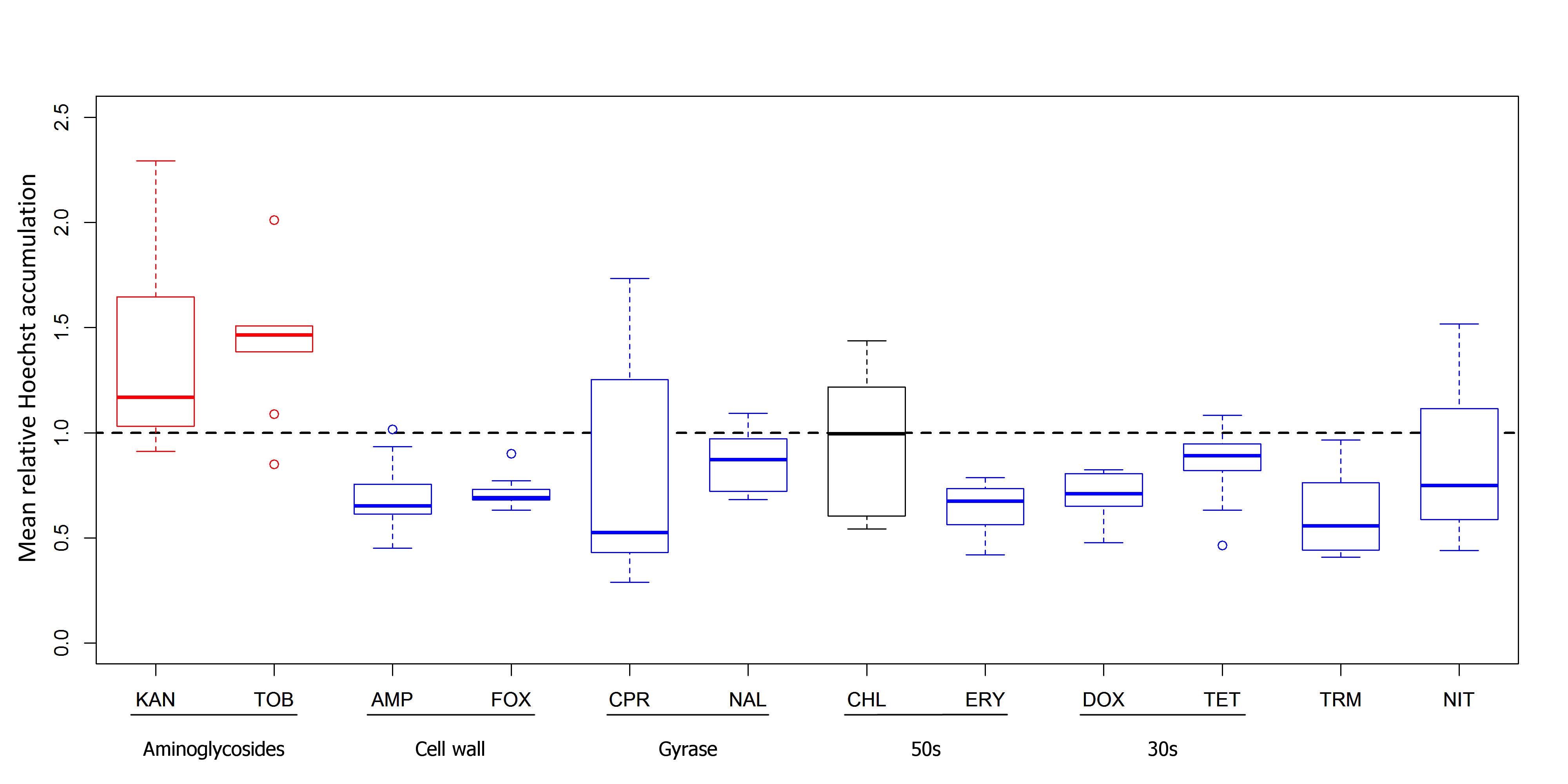


## Supplementary Figure S5 Membrane permeability (Hoechst dye) changes in populations adapted towards gradually increasing antibiotic concentrations.

For details, see Materials and Methods and Figure 4B, main text. For antibiotic abbreviations, see Table 1, main text.


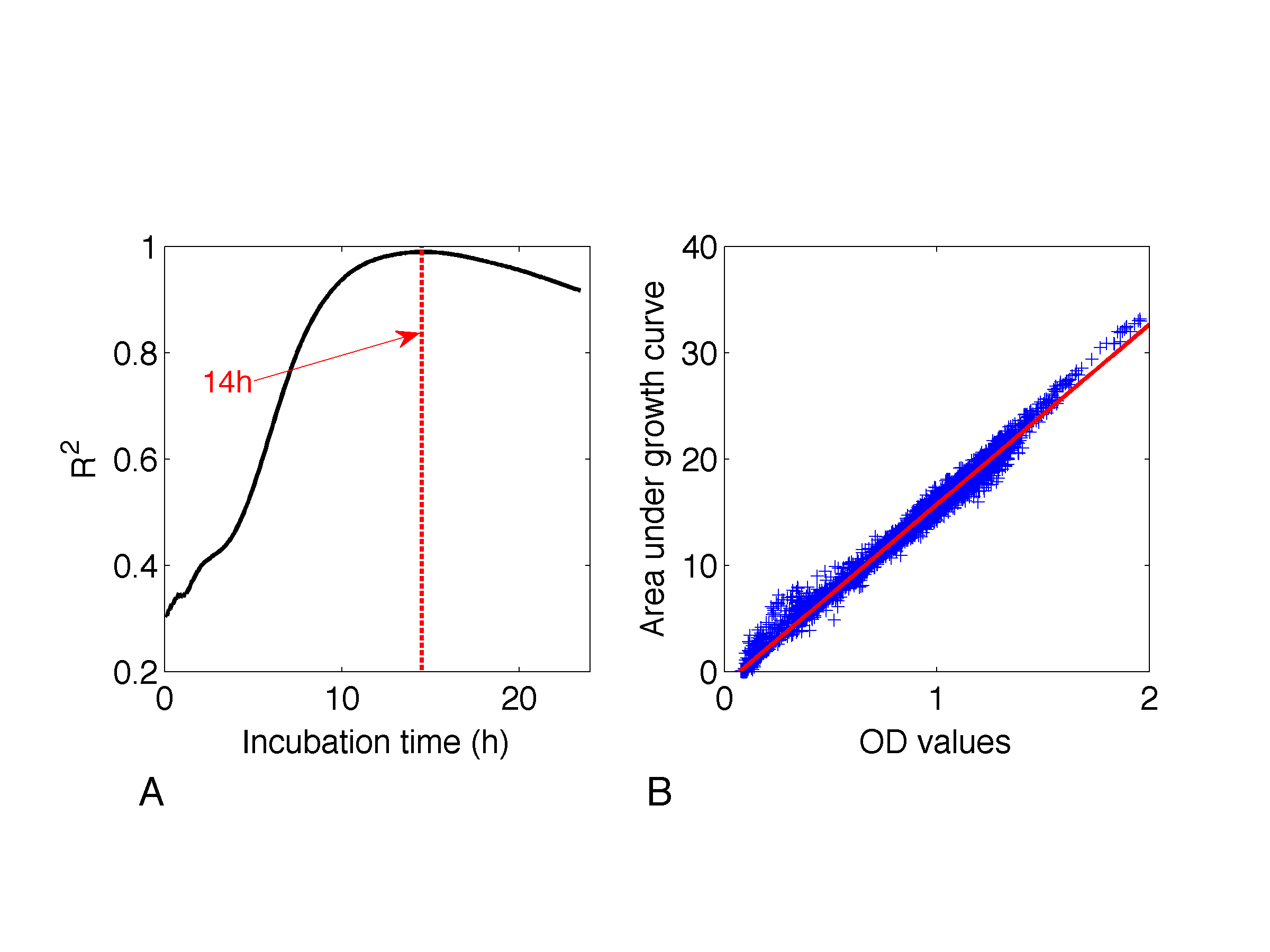


## Supplementary Figure S6 Optimization of the high-throughput fitness measurement assay.

To explore whether optical density measurements of liquid cultures at a single time point can robustly capture growth inhibition in the presence of antibiotics, we monitored growth of wild-type E. coli under various antibiotic stresses. Parallel cultures of E. coli were grown in 10 384-well microtiter plates, each being exposed to a different antibiotic at around IC_50_ concentration. Plates were shaken and incubated in a Synergy 2 plate reader. Optical density (OD600) was measured every 60 minutes for 24 hours and raw OD values were transformed as explained in Supplementary Methods. The area under the growth curve was calculated using custom made MATLAB scripts. For several different end timepoints (10, 12, 14, 20 hours etc.), we asked how the last measured OD values can predict the areas under the growth curves (384*10 = 3840 datapoints) using a linear regression model. Figure a) shows the squared correlation coefficients (R^2^) of linear fit as a function of reading time. A higher R^2^ value indicates a better fit between measurement at a single time point and measurement of the entire growth curve. The optimal incubation time (14h) is the one that maximizes the R^2^ value of the linear fit.  b) The OD values derived from the optimal incubation time point (14h) were plotted versus the values of Area Under Growth Curve. The linear regressor is indicated by the red line. The linear fit was carried out using all 3840 well data from the ten plates.

# Supplementary Tables

## Supplementary Table S1 Estimated MIC changes following the laboratory evolution towards gradually increased antibiotic dosage

| Applied antibiotic | Number of transfers | Number of generations | MIC change |
| --- | --- | --- | --- |
| ampicillin | 30 | ~ 240 | 20-fold |
| ciprofloxacin | 48 | ~ 384 | 328-fold |
| tobramycin | 45 | ~ 360 | 146-fold |
| trimethoprim | 36 | ~ 288 | 43-fold |
| erytromycin | 33 | ~ 264 | 28-fold |
| tetracycline | 39 | ~ 312 | 65-fold |
| nitrofurantoin | 30 | ~ 240 | 20-fold |
| nalidixic | 48 | ~ 384 | 328-fold |
| cefoxitin | 48 | ~ 384 | 328-fold |
| kanamycin | 45 | ~ 360 | 146-fold |
| chloramphenicol | 48 | ~ 384 | 328-fold |
| doxycycline | 30 | ~ 240 | 20-fold |

## Supplementary Table S2 Raw dataset of collateral sensitivity interactions identified at the level of antibiotic pairs.

Provided in a separate Excel spreadsheet.

## Supplementary Table S3 Decrease in minimum inhibitory concentrations of aminoglycoside-adapted lines.

We first selected 8 clones evolved in the presence of a single aminoglycoside in the lab (kanamycin, tobramycin, streptomycin). High-throughput analyses confirmed that these strains show collateral sensitivity towards several, overlapping classes of antibiotics (Supplementary Table S2). MIC changes were measured selectively for these antibiotics. For abbreviations, see Table 1, main text. Relative susceptibility is calculated as (MIC mutant) / (MIC control line).

| Lines adapted to this antibiotic | Type of evolutionary experiment | Antibiotic tested against | Relative MIC change |
| --- | --- | --- | --- |
| STR5 | fix sublethal antibiotic concentration | NAL | 0.44 |
| STR5 | fix sublethal antibiotic concentration | SLF | 0.56 |
| STR5 | fix sublethal antibiotic concentration | TET | 0.50 |
| STR5 | fix sublethal antibiotic concentration | DOX | 0.50 |
| TOB7 | fix sublethal antibiotic concentration | DOX | 0.49 |
| TOB7 | fix sublethal antibiotic concentration | CHL | 0.55 |
| TOB7 | fix sublethal antibiotic concentration | TET | 0.45 |
| KAN4 | fix sublethal antibiotic concentration | ERY | 0.25 |
| KAN4 | fix sublethal antibiotic concentration | NAL | 0.22 |
| KAN4 | fix sublethal antibiotic concentration | SLF | 0.30 |
| KAN4 | fix sublethal antibiotic concentration | CHL | 0.75 |
| STR10 | fix sublethal antibiotic concentration | CHL | 0.57 |
| STR10 | fix sublethal antibiotic concentration | ERY | 0.78 |
| STR10 | fix sublethal antibiotic concentration | SLF | 0.30 |
| STR10 | fix sublethal antibiotic concentration | TET | 0.45 |
| STR10 | fix sublethal antibiotic concentration | NAL | 0.38 |
| KAN4 | gradually increased antibiotic concentration | CPR | 0.39 |
| KAN4 | gradually increased antibiotic concentration | DOX | 0.24 |
| KAN4 | gradually increased antibiotic concentration | FOX | 0.27 |
| KAN4 | gradually increased antibiotic concentration | NAL | 0.21 |
| KAN4 | gradually increased antibiotic concentration | SLF | 0.18 |
| KAN4 | gradually increased antibiotic concentration | CHL | 0.36 |
| KAN4 | gradually increased antibiotic concentration | LOM | 0.50 |
| KAN4 | gradually increased antibiotic concentration | TET | 0.24 |
| KAN9 | gradually increased antibiotic concentration | CPR | 0.55 |
| KAN9 | gradually increased antibiotic concentration | FOX | 0.78 |
| KAN9 | gradually increased antibiotic concentration | NAL | 0.62 |
| KAN9 | gradually increased antibiotic concentration | DOX | 0.50 |
| KAN9 | gradually increased antibiotic concentration | SLF | 0.45 |
| KAN9 | gradually increased antibiotic concentration | CHL | 0.45 |
| KAN9 | gradually increased antibiotic concentration | TET | 0.49 |
| TOB3 | gradually increased antibiotic concentration | CPR | 0.60 |
| TOB3 | gradually increased antibiotic concentration | CHL | 0.28 |
| TOB3 | gradually increased antibiotic concentration | LOM | 0.86 |
| TOB3 | gradually increased antibiotic concentration | DOX | 0.33 |
| TOB3 | gradually increased antibiotic concentration | NAL | 0.44 |
| TOB3 | gradually increased antibiotic concentration | SLF | 0.36 |
| TOB3 | gradually increased antibiotic concentration | TET | 0.49 |
| TOB4 | gradually increased antibiotic concentration | CPR | 0.10 |
| TOB4 | gradually increased antibiotic concentration | CHL | 0.36 |
| TOB4 | gradually increased antibiotic concentration | LOM | 0.40 |
| TOB4 | gradually increased antibiotic concentration | DOX | 0.24 |
| TOB4 | gradually increased antibiotic concentration | NAL | 0.24 |
| TOB4 | gradually increased antibiotic concentration | TET | 0.24 |
| TOB4 | gradually increased antibiotic concentration | SLF | 0.27 |

## Supplementary Table S4 SNPs identified in aminoglycoside-adapted populations.

Provided in a separate Excel spreadsheet.

## Supplementary Table S5 Genes/protein complexes mutated multiple times in aminoglycoside adapted lines.

For antibiotic abbreviations, see Table 1, main text.

| Category | Complex\Gene | # lines carrying mutation | Description* | Clinical or experimental evidence supporting antibiotic resistance |
| --- | --- | --- | --- | --- |
| protein biosynthesis | fusA | 12 | Elongation factor G | The reduced aminoglycoside susceptibility in small colony variants of Staphylococcus aureus fusA mutants is caused at the level of antibiotic binding to the ribosome ([Norstrom et al, 2007](#ENREF_7)). |
| protein biosynthesis | rpsL | 5 | 30S Ribosome subunit. | A number of mutations in the rpsL gene encoding the S12 polypeptide generate resistance to streptomycin ([Springer et al, 2001](#ENREF_10)). |
| membrane protein, transport | cpxA | 2 | Sensor kinase of the CpxAR two-component signal transduction system. | The involvement of the Cpx system in antibiotic-mediated cell death through the Transcriptional regulation of mistranslation induced membrane stress ([Kohanski et al, 2008](#ENREF_5)). |
| membrane protein, transport | mlaD | 6 | MlaD predicted substrate binding protein of the phospholipid ABC transporter. | It prevents phospholipid accumulation in the outer leaflet of the outer membrane ([Malinverni & Silhavy, 2009](#ENREF_6)). |
| membrane protein, transport | pgsA | 2 | Phosphatidylglycerophosphate (PGP) synthase | Alterations in genes involved in membrane phospholipid biosynthesis influences antibiotic susceptibility ([Peleg et al, 2012](#ENREF_8)). |
| membrane protein, transport | potA | 3 | PotA is an ATP-dependent polyamine transporter. | The natural polyamine putrescine defenses against oxidative stress in E.coli ([Tkachenko et al, 2001](#ENREF_12)) |
| proton transport, aerobic respiration | ATP synthase complex | 4 | ATP synthase catalyzes the synthesis of ATP under aerobic cell growth. | Aminoglycosides generally require respiration for uptake ([Allison et al, 2011](#ENREF_1); [Taber et al, 1987](#ENREF_11)). |
| proton transport, aerobic respiration | Cytochrome bo terminal oxidase complex | 4 | These enzymes function as the major terminal oxidases in the aerobic respiratory chain of E. coli and contribute to the generation of a proton motive force (PMF). | Aminoglycosides generally require respiration for uptake ([Allison et al, 2011](#ENREF_1); [Taber et al, 1987](#ENREF_11)). |
| proton transport, aerobic respiration | NADH:ubiquinone oxidoreductase | 4 | NADH:ubiquinone oxidoreductase is an NADH dehydrogenase that catalyzes the transfer of electrons from NADH to the quinone pool in the cytoplasmic membrane and is able to generate a proton electrochemical gradient. | Aminoglycosides generally require respiration for uptake ([Allison et al, 2011](#ENREF_1); [Taber et al, 1987](#ENREF_11)). |
| proton transport, aerobic respiration | trkH | 8 | Potassium symporter, mutation close to another mutation known to increase K+ flux | A related regulator of K^+^ uptake has been shown to control both membrane potential and multidrug susceptibility ([Castaneda-Garcia et al, 2011](#ENREF_3)) |

## Supplementary Table S6 Functional enrichment analysis of SNPs.

Provided in a separate Excel spreadsheet.

## Supplementary Table S7 Pleiotropic effects of a single mutation in trkH

Relative susceptibility is calculated as (MIC mutant) / (MIC control line) where MIC is the minimal inhibitory concentration for the given antibiotic. The trkH mutation confers mild resistance towards aminoglycosides (gentamycin, kanamycin and tobramycin), but increases susceptibility to several other antibiotics. For antibiotic abbreviations, see Table 1, main text.

| Gene | Mutation inserted | Antibiotic to be tested against | Relative MIC change |
| --- | --- | --- | --- |
| trkH | Thr350Lys | TOB | 3.40 |
| trkH | Thr350Lys | GEN | >2.00 |
| trkH | Thr350Lys | AMK | 3.30 |
| trkH | Thr350Lys | STR | 2.60 |
| trkH | Thr350Lys | KAN | >2.00 |
| trkH | Thr350Lys | TET | 0.45 |
| trkH | Thr350Lys | SLF | 0.66 |
| trkH | Thr350Lys | NIT | 0.75 |
| trkH | Thr350Lys | NAL | 0.33 |
| trkH | Thr350Lys | DOX | 0.49 |
| trkH | Thr350Lys | CPR | 0.62 |
| trkH | Thr350Lys | CHL | 0.62 |

## Supplementary Table S8 Collateral sensitivity data on populations adapted to fixed, sublethal antibiotic concentrations

Provided in a separate Excel spreadsheet.

## Supplementary Table S9 Collateral sensitivity data on populations adapted to gradually increased antibiotic concentrations

Provided in a separate Excel spreadsheet.

# Supplementary Texts

## Supplementary Text S1 Processing of high-throughput bacterial growth data

We employed high-throughput optical density (OD) measurements in 384-well microtitre plates to monitor bacterial growth in the presence of various antibiotic treatments (see Methods, main text). As an initial step of our data analysis pipeline, we calibrated raw OD values by applying the transformation OD_calibrated_=OD+0.40449*OD^3^ to account for non-linear association between OD and cell density at high cell densities (parameters of the calibration formula were derived as in ref ([Warringer & Blomberg, 2003](#ENREF_13))).

The accuracy of high-throughput assays is often reduced by systematic errors (Malo et al. 2006). For example, slight variations in temperature or humidity within the plate during incubation may lead to local trends of altered growth (i.e. positional effects of wells within plates). Thus, we introduced normalization procedures to correct such systematic errors based on within-plate reference controls. To this end, we quasi-uniformly scattered 40 control wells on each plate containing a medium devoid of antibiotics and inoculated by wild-type cells. We used these wells both to set a baseline for maximum growth and to estimate and compensate for within-plate effects. Specifically, we used regression techniques to infer the OD value of untreated wild-type cells in each plate position based on the 40 control wells.

Because the shape of systematic errors might vary from plate to plate, we fitted a separate model for each plate. To better infer positional effects from a limited set of 40 control wells, we first characterized systematic errors by employing 16 control plates containing untreated wild-type cells in all 384 well positions and estimated hyperparameters for the regression model (i.e. few general model parameters that are assumed to be invariant across all plates). Observed OD values in each control plate are modelled by the sum of i) a constant OD value expected in the absence of any measurement bias or noise (‘theoretical OD’), ii) a linear trend capturing global linear row and columns biases within a plate, iii) a smooth surface capturing local and non-linear spatial biases (‘local spatial effects’) and iv) noise (‘experimental noise’) (Supplementary Figure 7).


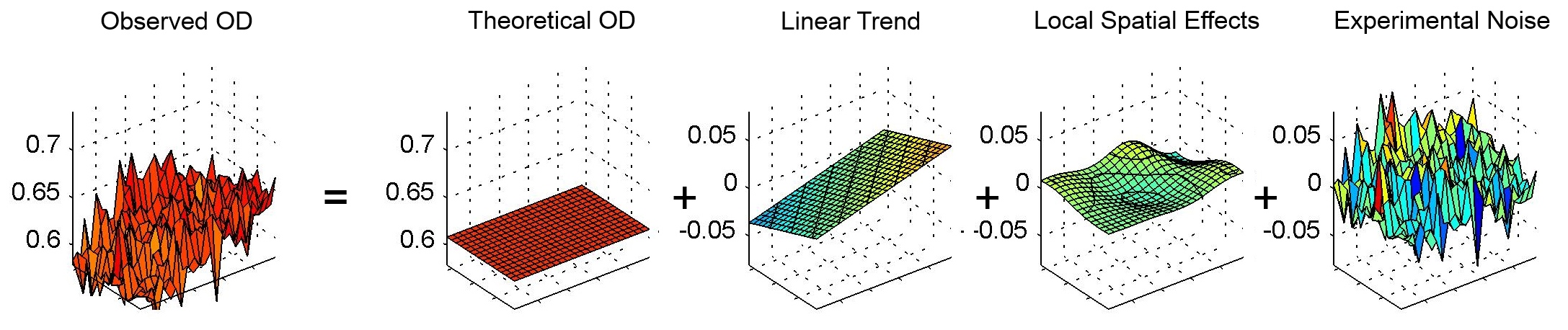


Supplementary Figure S7 Regression modelling of spatial effects in high-throughput growth assays.

Observed OD values of a plate containing untreated wild-type cells in all 384 positions are decomposed into a constant value, a linear trend, a smooth surface and random noise. See text for more details.

We modelled linear trends using linear regression, while local spatial effects and noise were estimated by Gaussian process regression with a squared exponential kernel ([Rasmussen & Williams, 2006](#ENREF_9)). We opted for Gaussian process regression because i) it is well suited to model non-linear spatial variation, ii) can jointly estimate both noise and spatial effects from the 40 control wells, and iii) preliminary experiments showed that the residuals remaining after linear trend elimination can be well captured by Gaussian process surfaces. The smoothness of the Gaussian process surface and the amount of residual noise were specified by 2+1 hyperparameters. These plate-independent hyperparameters were calibrated using the set of 16 control plates. Trend eliminations by linear fitting and Gaussian process regression were carried out in MATLAB (using functions regress() and gpr()([Rasmussen & Williams, 2006](#ENREF_9)), respectively).

Further measurements using 8 independent control plates demonstrated that both linear trend and local spatial effect corrections based on the 40 control wells substantially reduced measurements biases in the rest of 344 wells (Supplementary Figure 8). Thus, we fitted the above regression model to each antibiotic-treated plate to infer OD values of untreated wild-type cells at each plate position (OD_control_). Finally, we calculated a fitness score for each well as follows:

fitness = (OD – OD_t0_) / (OD_control_ – OD_t0_)

where OD_t0_ refers to the initial OD value of the well (maximum inhibition) and OD_control_ refers to the OD value of an untreated wild-type culture (maximum growth) as inferred by regression modelling.


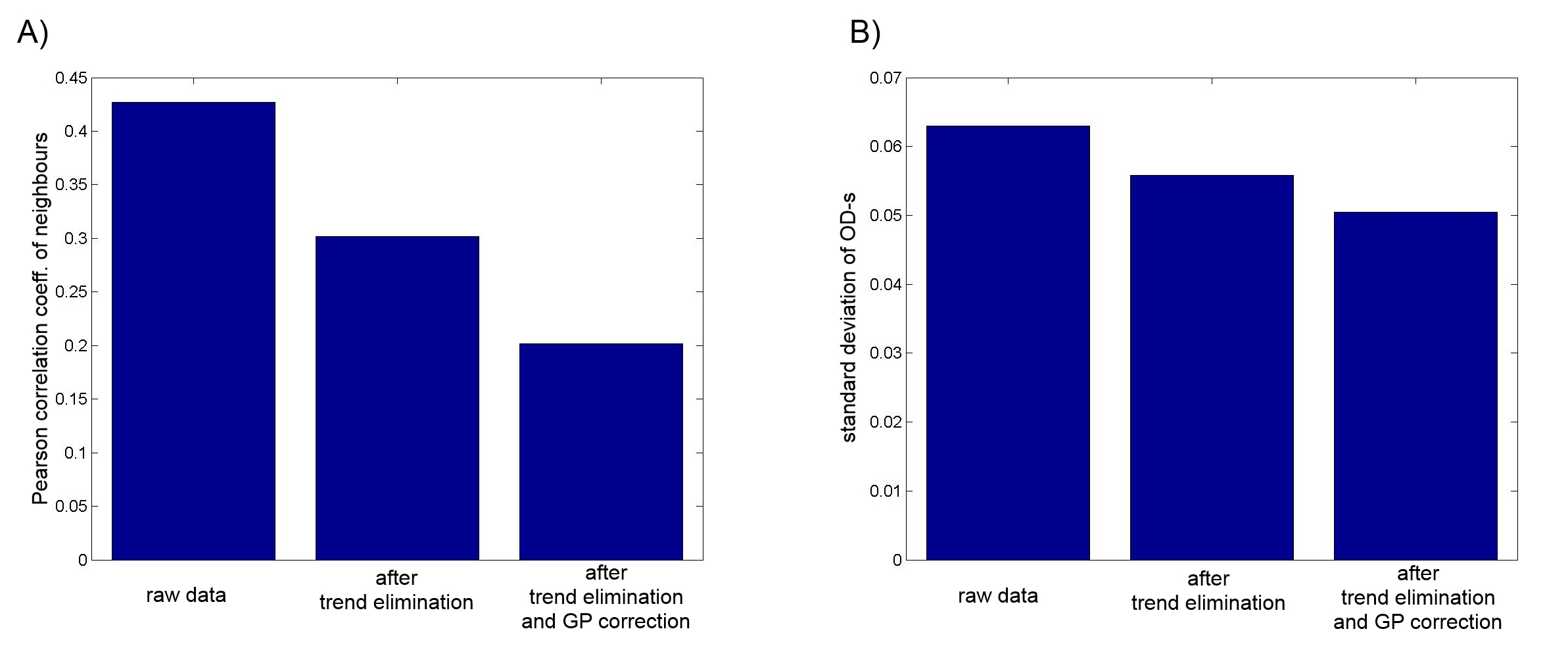


## Supplementary Figure S8 Normalization procedures reduce measurement bias and variation of control plate experiments.

Both linear trend elimination (‘trend elimination’) and Gaussian process regression-based correction (‘GP correction’) decrease the correlation of neighboring OD values (A) and standard deviation of replicate OD measurements (B) within plates. OD measurements were obtained from 8 control plates containing untreated wild-type cells in all 384 well positions.

## Supplementary Text S2 Accuracy of high-throughput interaction measurements and control for potential confounding factors

Here we address two questions related to the reliability of our collateral sensitivity interaction networks. First, we estimate the reproducibility of our high-throughput interaction measurement procedure. Second, we ensure that collateral sensitivity interactions are specific to antibiotic environments and not merely the consequence of a general fitness burden incurred by resistance.

*Estimating the reproducibility of evolutionary interaction screens*

First, we measured colony size on agar plates as an independent method to detect altered antibiotic susceptibility for a set of evolved lines. As shown in Supplementary Figure S1, relative fitness difference derived from high-throughput microtiter plate measurements correlates strongly with relative fitness difference derived from colony size measurements (Spearman’s rho = 0.745, P = 2.2*10^-16^, N=392 cases). A custom-built pipeline was used to segment and quantify colony size (i.e. area of colony) using the image analysis software CellProfiler ([Carpenter et al, 2006](#ENREF_2)). Second, we report that antibiotic susceptibility patterns of individual populations that had evolved under the same antibiotic pressure were generally very similar to each other (Supplementary Figure S2).

*Statistical procedure to control for the fitness cost of resistance*

We found a number of instances where the antibiotic-evolved line exhibited a growth defect in antibiotic-free medium, indicating costly resistance mechanisms. We set up a rigorous statistical procedure to ensure that the collateral sensitive interactions observed are not due to a general fitness decline. To achieve this goal, we first calculated the relative fitness difference (RF) of antibiotic-adapted and control populations: RF=(F_A_-F_C_)/F_C_ if F_A_<F_C_ and RF=(F_A_-F_C_)/F_A_ otherwise, where F_A_ and F_C_ are the fitnesses of antibiotic adapted and control adapted strains (i.e. those evolved in antibiotic-free medium), respectively. RF was estimated for all combinations of antibiotic-adapted populations and environments (including antibiotic-free medium). Low RF in antibiotic-free medium indicates a general fitness cost of antibiotic adaptation, while an especially low RF in a particular antibiotic-containing medium indicates collateral sensitivity. To control for potential biases in collateral sensitivity estimates that are due to general fitness costs, we compared RF values in antibiotic-free and antibiotic-containing media of all populations adapted to a given antibiotic. Wilcoxon Rank Sum test with false discovery rate correction procedure was used to determine whether the mean RF values in the presence of a given antibiotic are significantly lower than those measured in an antibiotic-free medium (all data available in Supplementary Tables S8-S9). This analysis was performed only for those combinations of antibiotics and sets of populations in which the evolved lines showed significantly lower growth than the adapted control lines under the same treatment condition (see details in Materials and Methods). Only cases with statistically significant differences (P<0.05 after FDR correction) were considered as collateral sensitivity interactions and retained for further analysis (Supplementary Table S8-S9, ‘p_values2’ sheet).

*Further evidence for the specificity of collateral sensitivity interactions*

The cost of resistance in antibiotic-free medium (in terms of a reduction in growth) was generally very low compared with the fitness loss in the presence of a specific antibiotic (Supplementary Figure S3). We conclude that the collateral sensitivity interactions detected in our screen are specific to the antibiotic environment and not merely the consequence of a general fitness burden incurred by resistance.

# References

Allison KR, Brynildsen MP, Collins JJ (2011) Metabolite-enabled eradication of bacterial persisters by aminoglycosides. Nature 473: 216-220

Carpenter AE, Jones TR, Lamprecht MR, Clarke C, Kang IH, Friman O, Guertin DA, Chang JH, Lindquist RA, Moffat J, Golland P, Sabatini DM (2006) CellProfiler: image analysis software for identifying and quantifying cell phenotypes. Genome Biol 7: R100

Castaneda-Garcia A, Do TT, Blazquez J (2011) The K+ uptake regulator TrkA controls membrane potential, pH homeostasis and multidrug susceptibility in Mycobacterium smegmatis. J Antimicrob Chemother 66: 1489-1498

Cressie NAC (1993) Statistics for spatial data New York: Wiley.

Kohanski MA, Dwyer DJ, Wierzbowski J, Cottarel G, Collins JJ (2008) Mistranslation of membrane proteins and two-component system activation trigger antibiotic-mediated cell death. Cell 135: 679-690

Malinverni JC, Silhavy TJ (2009) An ABC transport system that maintains lipid asymmetry in the gram-negative outer membrane. Proc Natl Acad Sci U S A 106: 8009-8014

Malo N, Hanley JA, Cerquozzi S, Pelletier J, Nadon R (2006) Statistical practice in high-throughput screening data analysis. Nat Biotechnol.24:167-75.

Norstrom T, Lannergard J, Hughes D (2007) Genetic and phenotypic identification of fusidic acid-resistant mutants with the small-colony-variant phenotype in Staphylococcus aureus. Antimicrob Agents Chemother 51: 4438-4446

Peleg AY, Miyakis S, Ward DV, Earl AM, Rubio A, Cameron DR, Pillai S, Moellering RC, Jr., Eliopoulos GM (2012) Whole genome characterization of the mechanisms of daptomycin resistance in clinical and laboratory derived isolates of Staphylococcus aureus. PLoS One 7: e28316

Rasmussen CE, Williams CKI (2006) Gaussian Processes for Machine Learning: MIT Press, .

Springer B, Kidan YG, Prammananan T, Ellrott K, Bottger EC, Sander P (2001) Mechanisms of streptomycin resistance: selection of mutations in the 16S rRNA gene conferring resistance. Antimicrob Agents Chemother 45: 2877-2884

Taber HW, Mueller JP, Miller PF, Arrow AS (1987) Bacterial uptake of aminoglycoside antibiotics. Microbiol Rev 51: 439-457

Tkachenko A, Nesterova L, Pshenichnov M (2001) The role of the natural polyamine putrescine in defense against oxidative stress in Escherichia coli. Arch Microbiol 176: 155-157

Warringer J, Blomberg A (2003) Automated screening in environmental arrays allows analysis of quantitative phenotypic profiles in Saccharomyces cerevisiae. Yeast 20: 53-67
